# Domestic Summary

**Published:** 1840-05-30

**Authors:** 


### DOMESTIC SUMMARY.

We learn that Dr. Edm. Du Barry, U. S. N. is preparing for the press a translation of "Bouillaud on Diseases of the Heart."

*Case of Sudden Death from Rupture of the Spermatic Vein.* By James McNaughton, M. D., of Albany.—For the early part of this case I am indebted to James P. Boyd, M. D. Dr. Boyd says:—"I saw Mrs. V——f, aged thirty-eight, January 28th, at 11 o'clock, A. M. She walked from her bed-room into an adjoining parlour when I called; told me that she had always enjoyed good health ; was naturally of a florid complexion, and healthy. The preceding night she did not rest well ; was troubled with dreams, and her bowels felt *bloated*, as she expressed it. She, however, rose, and took a hearty breakfast. Not long after, the uneasy sensations increasing, amounting even to pain, she took, at the suggestion of her husband, a dose of salts. This, however, soon produced nausea and vomiting, and the salts were rejected. The pain was so severe that I was sent for. I found her very pale, with a cold surface; lips and tongue appearing bloodless; complained of pain in the lower part of the bowels, with a constant bearing down, and some pain on pressure. She also complained of pain generally throughout the abdomen and thorax; difficulty of breathing and faintness; pulse at the wrist very small and weak; gave her a pill of four grains of calomel, and one of opium, to be repeated every hour until the pain was relieved, and to be followed by a dose of castor oil; left directions that I should be sent for if the pain increased. In a short time I was sent for in great haste; I returned about 12 o'clock, M., and found all the symptoms aggravated; the pain very severe, and principally confined to the lower part of the bowels; great faintness, requiring constant fanning; she became easy while I remained. Saw her again at 2 P. M.; and finding no reaction or abatement of symptoms, I proposed a consultation. Dr. B. P. Staats was called in, to whom I gave an account of the symptoms, and stated the embarrassment I experienced in relation to the true nature of the case. Some of the symptoms would lead one to suppose poison to be the cause, whilst the sudden and extreme prostration of strength, coldness of surface, and almost total extinction of pulse, would indicate the rupture of a blood-vessel; but then the pain could not be so well accounted for on that supposition. I supposed it hernia, but she declared repeatedly that she had no rupture or swelling about the abdomen. In confirmation of the probable correctness of this opinion, she did not refer the pain to any of the usual situations of hernia.

We met again at 4 P. M., when finding our patient no better, we proposed a thorough examination of the abdomen externally, and of the uterus per vaginam, which was accordingly done. During this examination Dr. Staats discovered an elastic, round tumour, near the umbilicus, one and a half or two inches in diameter. This we had no doubt was the cause of some part of her difficulty, although the patient declared that it was no such thing; that she had had that lump for fifteen or sixteen years—ever since her last confinement. At this stage of the case we concluded to call you into consultation, and the further history of the patient is known to yourself."

I saw the patient in the evening, about 8 o'clock, with Drs. Staats and Boyd; found her pale, cold, and almost pulseless; complained of pain in the lower part of the pelvis, and of an urgent desire to void urine. Dr. Staats had previously introduced a catheter, but very little urine was found in the bladder. I examined the abdomen; found the bowels slightly tumid, but not tense. There was general uneasiness of the whole abdomen, and great tenderness about the navel. Close to the navel I found a tumour, smooth, and rounded, which I had no difficulty in recognising as hernia. It was less in size than is stated by Dr. Boyd when first examined; it is therefore probable that it had been partially reduced before my visit. The patient complained excessively when I made even a gentle pressure on the tumour. Believing, however, that the tumour was the source of many, if not of most of the bad symptoms, and as the case was only of a few hours' standing, and no reaction had taken place, I thought it prudent and proper to return the protruded part into the abdomen. By gentle, steady pressure in the proper direction of the aperture, I succeeded in reducing the hernia in the course of a few minutes, the patient all the time complaining most bitterly of my hurting her. The reduction of the tumour was followed immediately by sickness at stomach and vomiting. Soon after which she became easier, and we left her with a pulse somewhat improved, after giving suitable directions about her treatment in our absence.

As there was cause to fear inflammation of the bowels in consequence of the long confinement of the protruded parts, an enema was directed in the evening, the stomach not admitting any cathartic by the mouth. In the course of the night of the 29th, Dr. Boyd was called to see her on account of the continuance of the pain at the lower part of the pelvis and throughout the abdomen generally. A prescription calculated to relieve urgent symptoms was made. We saw her again on the morning of the 28th, at 9 A. M. The skin was somewhat warmer about the hands, and the pulse more distinct; the face was pale, cold, and œdematous; bowels more full and more tender; at the umbilical foramen, upon careful examination, found a small, soft tumour, less than an inch in diameter, resembling an omental hernia, and scarcely distinguishable from the surrounding adipose matter, except by its circumscribed form. We still felt suspicious that this apparent protrusion had some agency in keeping up the urgent symptoms; but she expressed great dislike to my handling the swelling, charging me with all the bad feelings she had during the night.

In the course of an hour from this visit we met again, having been joined in the consultation by Dr. March. Upon a full examination of all the circumstances of the case, we came to the conclusion that the safest course would be to cut down upon the tumour, and ascertain its condition, rather than to irritate the part by an attempt at reduction by the taxis. The patient expressed a wish that Dr. March would operate. He proceeded immediately to the operation; the integuments over the tumour were thin—the tumour itself appeared round and smooth, covered by a sack, the vessels of which were dark and loaded with blood, but upon the division of this the enclosed adipose matter appeared of its natural healthy colour, and consisted of a cluster of masses of fat of different sizes. The umbilical foramen was narrow, but readily admitted a directory through it within the neck of the sack. The linea alba was divided downwards for a short distance by the bistoury guided by the directory, and the whole protrusion readily returned within the abdomen. The parts were then brought together in the usual manner, and the patient left to repose.

The operation did not afford any material relief, and the state of the protrusion was not such as would satisfactorily account for the intensity of the prostration. The bearing down and the urgent desire to void urine continued. The uterus was very low in the pelvis, and morbidly sensible to the touch, but not larger than usual in the unimpregnated state in persons who have borne child. In the evening the symptoms were somewhat milder; an enema had been exhibited, and came off without bringing much fœces with it. This was followed by a full dose of calomel, and the patient was left for the night to the charge of her nurse. On the morning of the 30th she seemed somewhat better,—skin warmer, and had a free discharge from her bowels. In the course of the day her distress returned, and she passed a restless, uncomfortable night. Saw her again on the morning of the 31st, when she was evidently sinking, and in the course of a few hours she died.

The case being singular in many respects, we expressed an earnest wish that a *post mortem* examination might be permitted, and the relatives, with great good sense, had the liberality to permit it.

*Appearances on dissection.*—The body was examined on the morning of the 1st of February, in presence of Drs. Staats, March, Boyd, Armsby, and myself. An incision was made from the ensiform cartilage to the pubis, through the linea alba, deviating to the left in the vicinity of the umbilicus, so as not to derange the relations of the parts in that vicinity. Well formed *pus* was found at the *umbilicus*, beneath the abdominal tendon, external to the peritoneum. Internally, the peritoneum appeared healthy, and free from inflammation. The tumour which appeared at the umbilicus, and which was reduced by the operation, proved to have no connection with the omentum, but to be merely a mass of adipose matter external to the peritoneum, filling up the umbilical foramen. Half an inch below there was a small opening in the linea alba, through which the herniary tumour, which had been reduced by the taxis, had protruded. The omentum, and the abdominal viscera in the immediate vicinity of the umbilicus, were healthy in appearance, and in their proper relative positions; but both the small and large intestines were enormously distended with flatus.

In this stage of the dissection, we were struck with the quantity of bloody serum which filled the interstices of the intestines, and proceeded to ascertain the state of the parts in the lower abdominal region and pelvis. We found, to our surprise, the *whole pelvis filled with a coagulum of venous blood*, which, when carefully removed, almost filled a large chamber pot. Upon carefully examining the abdomen and pelvis, we found the source of this fatal hæmorrhage. A tumour, about the size of a butternut, was found near the entrance of the fallopian tube into the uterus, and distant about two inches from that organ. It was first taken for the ovarium; but a more careful examination found the ovarium in its proper place, but shrivelled and hard. The same was the case with the ovarium of the left side. This copious hæmorrhage was now evidently from the right spermatic vein, and the tumour on the inner part of the fallopian tube was nothing more than a coagulum of blood, which, by a slight pressure, was readily removed. Those familiar with practical anatomy, know how enormously the spermatic veins are sometimes enlarged, even in the unimpregnated state of the uterus, and can therefore readily understand how suddenly the system would sink when they were ruptured. Having no valves, and communicating directly with the trunk of the vena cava on the right side, aided by the pressure of a high column of blood, the enormous loss found in this case, might have taken place in a very short space of time.

This appearance of things would satisfactorily account for the sudden sinking of the patient, and the want of reaction during the whole attack; but we could not thus account for the extreme tenderness of the abdomen, and particularly in the vicinity of the swelling, which appeared immediately below the umbilicus. The condition of the viscera in that vicinity did not indicate their having suffered any serious strangulation. Upon carefully wiping away the blood, however, from the *jejunum* and ileum, the upper part of the latter appeared dark for more than eighteen inches; and, upon drawing this portion between the fingers and thumb, it readily gave way, and had evidently lost its vitality. It was distinctly more friable than the same membrane either above or below this point. We concluded that this point had been protruded through the opening in the linea alba, and had been strangulated; that to this was attributable the tenderness about the navel, the vomiting, &c.; that the intestines were not thickened, nor the peritoneum inflamed more, might be accounted for by the exhausted state of the vascular system, not admitting of the occurrence of acute inflammatory reaction.

There is a case somewhat similar to this related in Professor Christison's work on Poisons.* In that case, however, there was a fal-

* Christison on Poisons, 3d ed. p. 107. For this reference I am indebted to my colleague, Prof. T. R. Beck.

lopian conception, and the hæmorrhage took place from the appendages of the ovum. In our patient there was no pregnancy, either uterine, or extra-uterine, and the patient had enjoyed good health for years previously.—*Transactions of New York Medical Society.*

---

*Case of Ozæna, accompanied by frequent paroxysms of Neuralgia Faciei, cured by the Extraction of a Tooth.* By CHAPIN A. HARRIS, M. D., Dentist, Baltimore.—Mr. S——, a resident of a neighbouring county, of a full habit, and slightly disposed to scorbutus, had, for a little more than two years, been the subject of an obstinate and distressing affection of the left nasal fossa, and of frequent attacks of pain, which he represented as being, at times, almost excruciating—commencing immediately over the first left superior molaris, thence shooting back to the angle of the jaw, then to the ala of the nose, inner angle of the eye, and not unfrequently to the top of the head. Ulceration had taken place in the mucous membrane of the affected nostril, and a thin, fœtid matter, occasionally streaked with pus and blood, was almost constantly discharged, excoriating the parts with which it came in contact. The cavity of the nostril had become so much closed by the thickening of its membranes, that the passage of air through it was prevented; the external integuments had assumed a dark florid appearance, and become considerably tumefied and sensitive to the touch.

His teeth having been suspected, though to all appearance perfectly sound, as having some agency in the production of the neuralgic affection, he was directed to a dentist to have them examined; but as none of them exhibited any signs of decay, it was thought to be dependent upon some other cause. Accordingly, the remedial means usually employed for this, as well as those for the other affection under which he was labouring, were prescribed; but from their use, although continued for several months, and under a variety of modifications, he derived no benefit.

His complaints becoming more and more aggravated, he at length became apprehensive as to their result, and determined, by the advice of his friends, to visit several of the medicinal springs in Virginia. At one of these he met with an eminent medical gentleman from one of the northern cities, whom he consulted; but neither from his prescription, nor the use of the waters of any of the springs that he visited, did he obtain the slightest relief; and, after remaining from home two months, he returned in a state almost bordering on despair.

To add to his affliction, he about this time began to be annoyed with a constant pain in the region of the antrum of the affected side. This, in connection with a soreness in a tooth immediately beneath, which he had felt throughout the whole course of his protracted and complicated disease, but which had not until now been sufficiently great to attract particular observation, soon led to the discovery of the cause both of the nasal and neuralgic affections, and also to the means by which they were finally cured. The pain in his jaw continuing to increase, and from its resemblance to toothache, he was induced, September 9th, 1839, to apply to me for advice. From the description which he gave of it, and the other circumstances connected with the case, the belief that the antrum was diseased, and that a morbid condition of some one or more of his teeth or their sockets had been chiefly instrumental in their production, at once forced itself upon me. With a view of satisfying myself more fully on this point, I gave his mouth a careful examination. His teeth, at least so far as their crowns were concerned, were all free from disease; but the socket of the first left superior molaris, which was that of the sensitive tooth, was considerably wasted—the tooth itself, particularly its outer and posterior surfaces, thickly coated with tartar, slightly loosened, and partially protruded from the jaw, whilst the surrounding gum was inflamed and spongy. The tooth having thus, as it would seem, from some cause or other, become obnoxious to the parts within which it was contained, and as it had no antagonist, its removal appeared to constitute the first and principal indication of cure. To this, upon its being advised, he readily submitted. The operation was followed by a sudden gush of thin, fœtid matter, from the antrum, which communicated with the socket of the tooth by an opening sufficiently large to admit of the easy introduction of the end of a small goose quill, and a subsidence of pain. The cause of his complicated malady was now revealed. The roots of the tooth were found to be greatly enlarged by exostosis.

The intervening transverse and longitudinal alveolar walls had been destroyed, and the place which they had formerly occupied filled with fungus. The edges of the surrounding wall were considerably wasted, and its surface interiorly rough and enlarged.

A strong solution of *argentum nitratum* having been applied to the diseased socket, by means of a camel's hair pencil, and the antrum syringed out with diluted tinct. myrrh, which last was directed to be repeated twice a day as long as the opening into that cavity should remain unclosed, the balance of the cure was entrusted to the restorative energies of the economy.

The following day he left the city, and I heard no more of him for six weeks; at the expiration of which time he again visited it, and called to inform me of the amendment that had taken place in his condition. He was now able to breathe through his left nostril almost as freely as the right—the discharge from it was greatly diminished, and of a more healthy character. He had had but one return of his neuralgic affection, which occurred the fourth day after the removal of the tooth, and was less severe than any of the former paroxysms. The opening into the antrum had closed, and the socket was rapidly filling with healthy granulation.

December 3d, I again had the satisfaction of seeing him, and of being informed that every vestige of his nasal and neuralgic affections had disappeared.

*Remarks.*—The circumstances connected with the history of the foregoing case would seem to justify the conclusion that the irritation produced by the enlargement of the roots of the tooth, had given rise to a morbid excitement in the mucous membrane of the antrum maxillary—that this had extended to that of the left nostril, where the parts being more exposed to external irritating agents, had taken on a new and more aggravated form of disease; and that the neuralgia was the result of the irritation in the nose, antrum, or socket, and most probably the last. How far the deposition of tartar that had formed on the tooth may have been accessory to the exostosis, is a question perhaps not easily solved. That it might produce such an effect can very readily be conceived, for when we take into consideration the morbid influence the presence of this substance frequently exerts upon the secretions of the mouth, the gums, and alveolar processes, it will not appear at all strange that it should give rise to this. The disease being dependent on inflammation of the periostia of the roots of the teeth, may be brought on, when favoured by a constitutional tendency, by any thing producing preternatural excitement in these membranes, and that salivary calculus often does this, is a fully recognised axiom in dental pathology. But how far it may have been concerned, either primarily or secondarily, in its production in this instance, I will not take upon myself to determine, inasmuch as there was one other circumstance connected with the history of the case that may have been the primary cause of the whole disturbance. That was, the want of an opposing tooth against which for this to act; and it may be well here to remark, that whenever this happens, especially to a superior molaris; and in the present case it had existed, as I was informed, for about seven years, the surrounding gum is apt to become inflamed, the periostium of its roots morbidly excited, and the socket to waste, and sometimes to become gradually filled with ossific depositions,* as though nature, conscious that the organ was of no further use, exerted her energies to expel it from the jaw. This tendency every dentist of observation and experience must have noticed; and Mr. Koecker, a distinguished European practitioner, in accordance with what would thus seem to be a law of the economy, recommends the extraction of all such teeth; but, as there are frequent instances where, by proper attention to their cleanliness, they may be permitted to remain with impunity, this advice should not always be followed.—*Maryland Med. and Surg. Journ.*

* The doctrine that teeth, after having lost their antagonists, are sometimes partially displaced by the gradual filling up of their sockets at the bottom with ossific matter, is denied by some; but the writer of this has met with several instances, as was clearly ascertained after the extraction of the teeth, where depositions of bone had actually taken place, and in five or six cases in considerable quantities. He has also conversed with several of his professional brethren, who say they have observed the same thing; and among the number, Dr. H. H. Hayden, of this city, a gentleman whose scientific attainments and professional acumen would render any deception in the matter altogether improbable.

The establishing of this fact, though somewhat irrelevant to the subject under discussion, was deemed necessary, in order to show the morbid effects that are frequently induced in the gum and socket of a superior molaris, by the loss of its antagonist.

